# Evaluation of cleaning and disinfection protocols for severe acute respiratory coronavirus virus 2 (SARS-CoV-2) on different hospital surfaces

**DOI:** 10.1017/ice.2021.23

**Published:** 2021-01-25

**Authors:** José Luis Barrios Andrés, María Justina Carriba Rodriguez, Maitane Aranzamendi Zaldumbide, Jose María Hernández, Margarita Viciola García

**Affiliations:** 1 Hospital Infection Control Division, Microbiology Service, Hospital Universitario Cruces, Barakaldo (Bizkaia), Basque Country, Spain; 2 Preventive Medicine Service, Hospital Universitario Cruces. Barakaldo (Bizkaia), Basque Country, Spain; 3 Virology Division, Microbiology Service, Hospital Universitario Cruces, Barakaldo (Bizkaia), Basque Country, Spain; 4 Commission on Infections and Antibiotic Policy, Department of Preventive Medicine, Hospital Universitario Cruces, Barakaldo (Bizkaia), Basque Country, Spain; 5 Preventive Medicine Service, Hospital Universitario Cruces, Barakaldo (Bizkaia), Basque Country, Spain

*To the Editor—*The coronavirus disease 2019 (COVID-19) pandemic has hit the world’s population harshly, causing severe illness that may require hospitalization and sometimes even intensive care unit (ICU) admissions.^1^ In fact, at certain times, a large proportion of patients admitted to hospitals have been infected by severe acute respiratory coronavirus virus 2 (SARS-CoV-2), becoming real reservoirs in the absence of proper hygiene and containment measures. The main route of transmission of SARS-CoV-2 is by droplets and aerosols; however, the role played by fomites remains unclear.^1,2^ Survival on different surfaces is estimated in hours and even several days.^2–5^ As an enveloped virus, SARS-CoV-2 is very sensitive to the usual disinfectants. However, in some hospital units, due to the high viral load that can be shed by patients, surfaces could be repeatedly contaminated over time, becoming a potential route of transmission.^6^

In this study, we evaluated the effectiveness of cleaning and disinfection procedures to eradicate the presence of the virus and therefore minimize the appearance of new sources of infection.

## Methods

From April 17 to June 4, 2020, 48 surface samples were prospectively collected in selected areas of University Hospital Cruces (Basque Country, Spain), a center with almost 1,000 beds that, during the study period, kept a median of 95 COVID-19 hospital patients per day, with a maximum of 165 and a minimum of 36 COVID-19 patients per day. The areas that were disinfected daily according to cleaning protocols established by the preventive medicine department of the hospital are listed in Table 1. The following samples were collected in clinical areas:

- 3 ICU boxes with COVID19 patients admitted for >5 days
- 1 ICU boxes after discharge of the COVID-19 patients
- 1 ICU box without patients (control)
- ICU warehouse with disinfected devices used in COVID-19 patients
- 3 general rooms with COVID-19 patients admitted >5 days
- 3 general rooms after discharge of the COVID-19 patients
- 1 general room without patients (control)

**Table 1.** Daily Cleaning Protocol Used in the Centre

Personnel who carry out the cleaning	Daily cleaning	Cleaning at hospital discharge	Used Compounds
Hospital cleaning service	• At least once a day (morning shift) • Furniture, floors, horizontal surfaces and bathrooms.	• Removal of curtains • Furniture, floors, horizontal surfaces and bathrooms. • Grids and windows	• Sodium hypochlorite (Clorosol gel 3%®)
Nursing Assistant Care Technicians	• At least once a day • Medical surfaces and equipment	• Medical surfaces and equipment	• Accelerated Hydrogen Peroxide Disinfectant Wipes:(Oxivir Excel Wipe®) or • Quaternary ammonium +/- biguanide Disinfectant wipes:(Cleanisept® Wipe Maxi and Sani-Cloth® AF Universal)

^a^Samples in COVID19 patients´ rooms-boxes were collected at different times: pre-cleaning or before the daily cleaning (about 24 hours after the last cleaning), and 3 hours later (post-cleaning).

Samples obtained from potentially contaminated objects were: pillows (n = 3), bed rails (n = 7), toilets (n = 6) and bedside tables (n = 8) in the COVID general rooms and bed rails (n = 5), shelves (n = 6) and medical equipment (n = 7) (infusion pumps and monitors) in ICU boxes. To complete the study, samples from the ICU warehouse (where the equipment is usually stored) were also collected.

Samples were obtained with sterile gauze pads (70% viscose and 30% Texpla polyester) previously moistened in universal virus transport medium (UVTM). Gauze pads and transport media were pretreated in Vivaspin 6 mL ultrafiltration tubes (Sartorius AG, Göttingen, Germany) by centrifugation at 3,000 rpm for 5 minutes. Polymerase chain reaction (PCR) detection of specific genetic regions of SARS CoV-2 virus (*N, Orf1ab* and *S*) was carried out in the QuantStudio 5 equipment (both from Applied Biosystems, ThermoFisher Scientific, Waltham MA).

## Results

No viral RNA fragment was detected in any of samples collected in the pre- or postcleaning samples from any of the studied areas.

## Discussion

We evaluated the effectiveness of disinfection measures implemented in our center to eradicate, or at least reduce, the presence of the virus. For this purpose, unlike other studies, we performed sample collection with sterile gauze, dragging to collect a large amount of representative material. We also used a molecular detection technique that combines various targets of virus genome to improve the sensitivity. Additionally, we extended collection time frame to increase the probability of detecting presence of the virus. Nonetheless, we did not detect the virus in any of the studied areas.

Detection of SARS-CoV-2 on hospital surfaces varies greatly.^1,7^ According to some studies, it was frequently detected before cleaning or disinfection in COVID-19 patient areas and especially on objects with greater handling^1,2,4,8,9^; in other studies, it was only detected in isolation areas and sporadically after cleaning or disinfection.^4,10^

In our case, the main difference is that the virus was also not detected before cleaning and disinfection, even having been carried out just once a day, increasing the likelihood of surfaces being contaminated. We believe that 2 factors could have influenced the absence of detection. On one hand, the thoroughness and way of carrying out the cleaning and on the other, the remaining effect of the compounds used may have prevented contamination. The cleaning was carried out, in all cases, applying a unidirectional wave friction protocol that achieved a total drag of the material deposited on the surface. On the other hand, different compounds were used in disinfection: 3% sodium hypochlorite, accelerated hydrogen peroxide 0.32%, and quaternary ammonium compounds 0.5% with biguanide. The compound itself does not seem to be of great importance because SARS-CoV-2 is extremely susceptible to the disinfectants usually used. Instead, the format of the product might have been influential; sodium hypochlorite was used in gel form and hydrogen peroxide and quaternary ammonium, with or without biguanide, in wipes even containing surfactants in some cases. Our hypothesis is that these compounds could have had a certain postadministration duration effect that would have minimized the possibility of subsequently detecting the virus on the surfaces studied.

In conclusion, these results indicate that the cleaning protocol used in our center is completely effective in eradicating the virus from the surfaces and medical devices most likely to be colonized, improving safety of patients and their environment.^6^
